# Abstracts and Gleanings

**Published:** 1883-10-20

**Authors:** 


### ABSTRACTS AND GLEANINGS.

**Country and City Doctors.**—We extract the following from an article on this subject in the Medical and Surgical Reporter:

A "new physician," who had been trying city practice for something more than a year, was at last housed in nearly three months by illness. On meeting him one day afterwards, I asked what had been the matter? His answer was a volume compressed into an utterance of outraged feeling: "Nothing but bad bills! It was nothing but general exhaustion from running after bad bills—and mental worry—they wore me out with faithless promises."

Physicians in the country are sometimes prone to think that city doctors do not have to work hard for their money, and the former conclude to remove to the city for an easier field. Unfortunately these often find the field so easy that it becomes necessary to move back again. The writer has lived in his present location eleven years. During that time at least a score of doctors have located in the same neighborhood, tried it a few months or years, then moved away. Several of these were from the country, men of good practice there, but almost clientless here. In the course of a year or two several wisely returned to their old neighborhoods, where they were known, and doubtless where they were welcomed by appreciating friends—for nowhere does a man feel so lonely and helpless as in the wilderness of a great city of strangers. Because ten physicians succeed in making a living, with their offices near each other on a certain street, it is a grave mistake to expect that the prospect promises as much to twenty more because they put out their shingles. A few months ago I counted the signs of more than thirty doctors in the space of seven squares on this street and the immediate cross-streets. One has been removed by disease, one removed himself by suicide, six have sought fatter pastures elsewhere.

Physicians themselves are not always to blame for these mistakes of judgment, especially if they come from the country. Too often they are easy game in the agile hands of adroit real estate agents, who so well know how to praise up the prospects of any neighborhood where they may have a house to let or to sell. To illustrate: Some years ago a well-to-do physician from a large interior town thought he would like to move to this city; found a desirable up-town neighborhood where considerable building was in progress, and consulted a local real estate agent that had houses to rent. The astute agent soon furnished most satisfactory assurances of the wisdom of the doctor's project, and clinched his faith by citing the case of another doctor who had lately located in the same neighborhood, and "succeeded so well that in a year's time he was able to buy a $7,000 house!" Of course, the willing doctor rented a house, moved to the city; in six months discovered he had made an unprofitable venture; tried another part of the city for six months more, then moved back to his old home—a year and two thousand dollars out, besides business missed.

But does the city doctor have the easier life? No. One hundred dollars in the country will reach as far as three hundred to six hundred in large cities, according to location. With fees nearly the same among the general rank and file of the profession, it is self-evident that the city doctor must do extra work, must sustain extra wear and tare of body and mind to come out even. The perplexing uncertainties of a city establishment harass the lives of city physicians by day and by night. Hence, also, that so many city practitioners feel impelled to round out limited receipts by engaging every spare hour, needed for rest and recuperation, in the severe toils of authorship in some form. It is the pressure of current expenses rather than the pressure of overwork, that keeps so many pens busy turning out reports, reviews, essays, and books; and hence the publication of so much that is speculative, artificial, unreliable, and unsatisfying to the mass of practitioners. To practitioners in general in cities, professional experience is not so rich and varied as in country locations. The hospitals, infirmaries and dispensaries in cities, run by a select few to foster reputation in a specialty, or to open paths of introduction to general practice, monopolize the great harvest of cases by which beginners are usually enabled to gain prompt foothold and win distinguished success. In cities not the poor only, but many in entirely comfortable circumstances, become inmates of hospitals for treatment in cases of accidents, or special diseases, or they receive "out-door" treatment at their homes through dispensary ministrations; while in country locations all the surgery, obstetrics, eye and ear cases, skin and venerial complaints, and the general scope of medical observation and treatment come fully under the study of the local practitioners, thus immensely expanding their opportunities for securing broad and rich experience and early recognition of professional skill.

A physician's prosperity must be gauged at last not by what he is compelled to spend from year to year, but by what he is enabled to save above the current out-go. Ostentation is not wealth. The necessity for display is an exacting task-master.

As compared with large cities, the advantages of country locations afford the great mass of physicians much better and safer fields for experience and practice, for professional and social appreciation, for mental and moral expansion, for lucrative reward and substantial prosperity. Among his unpretentious, honorable peers, the honors of his attainments and office make the worthy physician in the country a head above men—a light to look up to in the community: in the rabble of great cities the physician is too often a head below men—a glimmering luminary lost in the jostle and confusion of lights.

"I don't see how all these doctors *live!*" remarked a moneyed business man to another, within my hearing, on a down-town street. During a few minutes' conference on the sidewalk between these lords of ample means, two physicians had jogged past in their peculiar calash-top vehicles, that here announce the professional calling.

"The half of them go with less than half a living," remarked the one addressed, as they moved down street together.

Such derisive flings are not made against the respectable physician in country places, where the public know that he has an ample exchequer, and besides the income of a good practice, owns a good farm or grist mill, or several town properties, acquired by his frugal management of modest yearly profits. In the country such is the general possibility; in the city, the rarest of exceptions.

It was the testimony of a successful, prominent Philadelphia physician, on withdrawing from practice some years ago, that in an experience of a quarter of a century, as his income from practice gradually crept up from six hundred a year to three thousand dollars, the expenses of his advancement and social exactions kept even pace with his earnings, and left him nothing to lay by for future dependence.

And it need not be imagined by physicians in the country that eminent college professors acquire fortunes by practice: many of the greatest and the best have died insolvent. In the accessible calm and relaxation that physicians find in country locations without detriment to pocket and prospects, there is a luxury of comfort unknown to the profession amid the almost pauseless din and hurry of city life. *There* may he always find equivalent substitutes for money, when money there is none; but such is not the case in cities. *There* he may be greeted by refreshing landscape, expansion of scene, quiet for weariness, reflection and sociality; but not so in cities where all is contracted, walled-in, hustle and tussle, with no rest to eye or ear, or nervous system, or head or heart from year to year. Is it any wonder so many long to get away for even a little while to enjoy the restful peace of nature's quiet in God's country?

**Strychnine.**—Dr. J. A. McCorkle ("Pres. of the Med. Soc. of the County of Kings," Dec., 1882) calls attention to a fact often overlooked, namely, that stimulants like strychnine, used very long or in very large doses, ultimately have a depressing influence and exaggerate the conditions for which they were originally prescribed. The author suggests that physicians should be careful that their patients do not continue a tonic too long. Such medicines should be given in such doses as are calculated to raise the depressed functions to a normal standard; as soon as the physiological condition is approached, the dose of the tonic should be gradually diminished, and finally stopped altogether when health is restored. The too long continued administration of a tonic like strychnine depresses the nervous system just as over-stimulation of a nerve by electricity exhausts irritability. In the same way a bitter tonic may have relieved indigestion by stimulating the vascularity of the gastric mucous membrane; but the same tonic, given too long, may produce gastric catarrh, and bring on disturbances of digestion worse than those which had been originally relieved through its use.

Strychnine has been advocated as a respiratory stimulant by Fothergill; it is believed that, if used too long, it may depress res piration and interfere with the oxidation of sugar in the lungs to such an extent that temporary glycosuria may result. In support of this, cases are on record where sugar has been observed in the urine of those who had been taking nux vomica for a considerable period. The author believes that strychnine, in combination with phosphoric acid, is the best respiratory stimulant we have, but that it may do great harm if continued too long. He also believes that it has decided curative powers in diabetes mellitus, but that this disease is still too little understood to permit of our using strychnine in the treatment of it; for we can not decide when we are doing good and when we are doing harm.—*N. Y. Medical Journal.*

**Blood-Letting.**—Dr. McCulloch in the Journal of the American Medical Association, writes :

Do not the present generation of physicians abuse their patients by their "anti-exhaustive treatment," and by their stimulating, brandy and milk course do more harm than ever the lance did ? Why, thousands of patients at the present day, after a protracted illness, come out of the hands of the doctor confirmed inebriates, fit only for bar-room loafers.

Women in former days were bled for headache, backache, pains real or imaginary, and they grew fat, raised large families of healthy children, and lived to a ripe old age.

But in our day the hypodermic syringe has taken the place of the lance, and for their pains and aches they are chucked full of morphia daily. Sensibility deadened, nervous system unstrung ; muscular system relaxed, and they become fit subjects of abortion or premature labor. Their children dwarfed and they die at a premature age.

Would it not be better if we would follow more the teachings of nature on this subject ? Why, one-half of the human family, and the best half at that, by a fixed law of nature, which is God's law, lose from six to ten ounces of blood monthly, and continue to average so for 30 years, being bled not less than 360 to 400 times in their life, for what purpose ? To carry off the surplus blood, like a safety valve, to relieve their congested organs.

And yet if a man has double pneumonia, lungs congested and pressed upon with a pressure of 100 pounds to the square inch, breathing 60 times to the minute, you are afraid to take a pint of blood from him for fear he dies from exhaustion, and you chuck him full of quinine, iron, brandy and milk to support him, and morphia to relieve his pain ; and your man dies and you console yourself by the reflection that you gave him the best chance for his life by using the latest and best treatment approved by the profession.

I have, in a continuous practice of over 35 years, met with some cases of pneumonia, and I never had a case of primary inflammation of the lung in the adult but I bled freely in the congestive stage, and although I say it myself, it was very rarely that they did not recover rapidly after one or two decided venesections.

I had an attack myself, in the spring of 1858, of acute pneumonia of the left lung. I was bled twice from the arm, cupped and blistered, and I made a speedy and good recovery. I can remember what a load was taken off my chest at the first bleeding; how the pain ceased; and how easily I breathed; and with a full dose of morphia how well I slept that night. In the last few years, since bleeding as a remedy has been abandoned, nearly all the cases I have seen have been in consultation with other physicians, and the great majority of them were very bad subjects, men who were broken down by strong drink. I saw them in the advanced stage, when the time for bleeding had gone by, if at all admissable in such subjects—seven in number. They all died promptly, full of morphine, quinine, iron, brandy and milk.

Dr. John L. Atlee, our retiring president, in his address to the members of the American Medical Association, at Cleveland, June 5th, last, said:

"I feel well assured that the almost total disuse of the lancet has cost many valuable lives. From a very large experience in its use I am satisfied, *fully satisfied*, that if we depended more on the early use of the lance, in the congestive and inflammatory states of many diseases, our practice would be made more successful than it now is. It is, in my opinion, a very important subject, and I feel assured that ere long the lancet will be more freely used than it is now."

When I heard these words fall from his venerable lips, an old veteran in the profession, brim full of knowledge and wisdom gathered from scientific research and an experience of 60 years of active practice in the profession, although not a Methodist, I could scarcely restrain myself from shouting, Amen!

Dr. Davis, of Chicago, says there was, during the year 1882, as per the census of 1880, one death in the city of Boston to every 532 of the population; 1 to every 579 in Chicago; 1 to every 441 in San Francisco; 1 to every 1,088 in New Orleans. He says sanitarians should investigate the cause, and suggest some means of checking this fearful mortality. Certainly this is good advice, and upon the principle that "an ounce of prevention is worth a pound of cure," it is well-timed. But I say to you, fellow-practitioners, clean up your old rusty lancet, and you that have none buy one; carry it with you to the bedside of the sick, and when you meet an enemy so formidable as eclampsia or pneumonia, stand in the advance guard strike with your lance one or two decisive blows in the onset of the conflict, and it will do more toward subduing the enemy than all the stimulating, nourishing treatment of the present day. "Quit yourselves like men."

[We endorse the above in toto.—SOUTHERN MEDICAL RECORD. W.]

**Neuralgia.**—Neuralgia of the eye is often speedily cured by full doses, 10 to 15 grains of the muriate of ammonia repeated at intervals of two to four hours. We have seen two desperate cases of this kind where the eye appeared as if attacked with the severest inflammation and the pain atrocious, yielded promptly to this remedy.—*Ga. Ec. Med. Journal.*

**The Physiological Effect of Coffee.**—Dr. J. A. Foot, of Rio de Janiero, (Bull. Gen. de Therap., June 30.) Dr. Foot gives us the effects of a strong dose of coffee upon his own person after recording his condition for fifteen days of total abstinence from coffee, and follows his record of the effects of the strong dose, by noting the influence of two cups of coffee daily for twenty-five days. The most interesting part of his paper is his record of the effects of the strong dose:

At the time of taking it, his pulse was 72 in the morning, reaching 84 during the day. He made an infusion of over ℥ viij of coffee in a quart of boiling water, drinking the whole of it during the day from 7 a. m. to 9 p. m. During that day the pulse increased in rapidity to 108 in the afternoon; in the evening it reached 114. He went to bed at 11 p. m., but could not sleep, reflex contractions were produced in nearly every part of the body alternately. Very painful cramps in the thighs, legs, feet, walls of the thorax and in the muscles of the hyoid region. These cramps persisted throughout the night, but moderated in severity on the following morning. The tongue was dry and there was a certain degree of constriction in the chest. At the same time there were frequent cramps in the stomach accompanied with nausea. The intestines were the seat of frequent borborygums, and of abundant liquid secretion which produced eighteen evacuations. The pulse kept between 110 and 112 through the night. It was intermittent, as was the heart's action, losing one pulsation to every four. The next day the pulse was 76, there was headache and no appetite.

In this experiment, then, the coffee acted on the organs and functions of the central cerebro-spinal system, producing insomnia by exciting the brain, producing the cramps in the muscles, pains in the stomach, disturbance of the intestines and of the heart by exciting the spinal cord, an excitation of the reflex force or excito-motor. He considers that this irritation affects equally the spinal roots of the sympathetic, and in paralyzing the vaso-motor nerves. In this way explanation is given of the cause of the excessive secretion from the intestine and of the abolition of sexual power.

His other experiments with moderate doses, prove to his satisfation, that the use of coffee does not prevent advanced age and the preservatfon of good health; and that life seems to be prolonged in countries where coffee is much used.—*Jour. Ame. Med. Association.*

**Effect of Alum Gargle upon the Teeth.**—M. Young prescribed a gargle containing a small proportion of alum for a woman suffering from chronic pharyngitis with catarrh of the middle ear. The patient, finding relief, continued its use for some three weeks. But perceiving that, at meals, her teeth began to crumble into little pieces, she consulted her dentist, who considered it due to the alum gargle, as when the enamel is removed from the teeth, the alum breaks down the dentine. To prevent this it is best, immediately after using an alum gargle, to wash the mouth cut with a solution of bicarbonate of soda or an alkaline water.—*Courier Medical.*

**Cannabis Indica; A Valuable Remedy in Hemorrhagia.**—In the British Medical Journal, May 26, 1883, Mr. J. Brown, of Bacup, observes:

"Indian hemp has been vaunted as an anodyne and hypnotic, having the good qualities of opium without its evils. Also in dysmenorrhœa and insomnia it has not proved of much benefit. The drug has almost invariably produced some marked physiological effect, even in small doses. Text-books give the dose as ten minims and upwards, but five minims is the largest dose that should be given at first. If bought from a good house, the drug is not inert or unreliable. A drug having such marked physiological action ought to have a specific use as a therapeutic agent. Indian hemp has such specific use in menorrhagia—there is no medicine which has given such good results; for this reason, it ought to take the first place as a remedy in menorrhagia, then bromide of potassium and other drugs. The *modus operandi* I cannot explain, unless it be that it diverts a larger proportion of blood to the brain, and lessens the muscular force of the heart. A few doses are sufficient; the following is the prescription:

"℞ Tincturæ cannabis indicæ.................... ♏ xxx,

Pulveris tragac. co.......................... ʒ j,

Spiritus chlof............................ ʒ j,

Aquæ ad................................. ℥ ij.

One ounce every three hours.

"Four years ago I was called to see Mrs. W., aged 40, multipara. She had suffered from menorrhagia for several months. Her medical attendant had tried the ordinary remedies without success. Indian hemp was given as above. Its action was speedy and certain. Only one bottle was taken. She was afterwards treated for anæmia, due to loss of blood. Twelve months after this my patient sent for a bottle of the "green medicine." I learned afterwards that she had sent this medicine to a lady friend, who had been unsuccessfully treated by another medical man for several months for the same complaint. It proved equally successful. The failures are so few, that I venture to call it a specific in monorrhagia. The drug deserves a trial. It may occasionally fail; this, however, is not to be wondered at in a complaint due to so many different causes, and associated with anæmia and other cases of plethora."

Robert Batho, M. D., M. R. C. P., Castleton, Isle of Man, writes in reference to the same subject: "Considerable experience of its employment in menorrhagia, more especially in India, has convinced me that it is, in that country at all events, one of the most reliable means at our disposal. I feel inclined to go further, and state that it is *par excellence*, the remedy for that condition, which, unfortunately, is very frequent in India.

"I have ordered it, not once, but repeatedly, in such cases, and always with satisfactory results. The form used has been the tincture, and the dose ten to twenty minims, repeated once or twice in the twenty-four hours. It is so certain in its power of controlling menorrhagia, that it is a valuable aid to diagnosis in cases where it is uncertain whether an early abortion may or may not have occurred. Over the hemorrhage attending the latter condition. it appears to exercise but little force. I can recall one case in my practice in India. where my patient had lost profusely at each period for years, until the tincture was ordered ; subsequently, by commencing its use, as a matter of routine, at the commencement of each flow, the amount was reduced to the ordinary limits, with corresponding benefit to the general health. Neither in this or in any other instance in which I prescribed the drug, were any disagreable physiological effects observed.

"I could say a few words in its favor, as to its action in allaying irritative cough, but I prefer confining myself to a point on which experience has left me no room for doubt."— *West. Laucet.*

**Paraldehyde and Acetal as Hypnotics.**—At a recent meeting of the Berlin Society of Psychiatry and Nervous Diseases Dr. Langreuter ("Deutsche Medizinal-Zeitung," Aug. 23, 1883) gave his experience with these drugs during a period of eight months at a lunatic asylum.

The paraldehyde was employed in the form of the following mixture :

| | | | |
|---|---|---|---|
| R | Paraldehyde........................ | 25-0 grammes, | |
| | Oil of peppermint.................. | 5 drops, | |
| | Olive oil, enough to make........... | 50-0 grammes. | M. |

The difficulty of dissolving the paraldhyde and its burning taste were noted as drawbacks to its employment. The usual dose was six grammes ; 2,300 grammes were used in all. Beyond a slight and transitory irregularity of the pulse, no abnormal phenomena were observed ; in quality, the pulse was always somewhat fuller. During sleep produced by the drug the breathing was deeper and slower ; the pupils were not generally so much contracted as in physiological sleep, but dilatation was in but few instances. Sleep ensued in from five minutes to half an hour. In two cases the patients fell from the chair within a minute after taking the dose. The sense of sleepiness was readily interfered with, as the speaker had proved in his own person ; quiet, therefore, favored the hypnotic effect, which took place after ninety per cent. of the evening doses, and after sixty-one per cent. of those given by day. In sixty-one per cent. a full night's sleep (seven or eight hours) was produced. A quieting influence was exerted even when sleep did not follow—most strikingly in excited paralytics and epileptics. In certain nervous affections, such as migraine, etc., the effect was favorable also.

The action of acetal was not so satisfactory, and only half as energetic, the medium dose required being from 8 to 10 grammes. Moreover, the taste and smell were found much more intense, and the effect was not so lasting. Nevertheless, excited patients became quieter, even if they did not fall asleep, contrary to what was observed after the use of chloral. In all 2,700 grammes were used. Out of one hundred and sixty-seven trials, seventy-five per cent. were successful. Six grammes of paraldehyde and ten grammes of acetal were each found equal to 2.5 grammes of chloral in activity. Although both the new drugs were dearer than chloral, they were to be preferred in certain cases in which the latter was contraindicated, as in cardiac affections. The speaker did not recommend acetal.—*N. Y. Med. Rec.*

**Treatment of Tonsillitis.**—Dr. Solis Cohen gives the following treatment, which he says is pursued at the Philadelphia Polyclinic with eminent success:

1. In simple inflammatory tonsillitis, take two fluid drachms each of the ammon. tinct. of guiac. and the co. tinct. of cinchona, mix with six fluid drachms of clarified honey and shake together until the sides of the vessel are well coated; add gradually a solution of eighty grains of chlorate of potassium in four ounces of water, shaking meanwhile. This to be used as a gargle every one-half to three hours. Relief is usually experienced within a few hours, and recovery is prompt. A saline cathartic may accompany the use of the gargle. None of the cases seen suppurated, and if seen within the first twenty-four hours such accidents are very unlikely.

2. In rheumatic or constitutional tonsillitis (characterized by intense pain in swallowing causing great accumulation of saliva from unwillingness to swallow, with slight, perhaps no, congestion of throat, and subsequent fever; one or both tonsils becoming enlarged after some hours as the febrile symptoms decline, and muscular or joint rheumatism sometimes developing later.) after a saline cathartic, give the following in tablespoonful doses every two hours:

| | | |
|---|---|---|
| ℞ | Sodi salicylat. . . . . . . . . . . . . . . . . . . . . . . . . . . . . . . . . . . | ʒ ii, |
| | Ol. gaultheriæ. . . . . . . . . . . . . . . . . . . . . . . . . . . . . . . . . . | *m* i, |
| | Liq. ammon. citrat. . . . . . . . . . . . . . . . . . . . . . . . . . . . . . | aa. ℥ ij |
| | Syrup simp. . . . . . . . . . . . . . . . . . . . . . . . . . . . . . . . . . . . | |

Lengthening the intervals as the pain subsides. Pieces of ice or the guiac gargle promote comfort, and the stiff neck is best relieved by faradization. Salicylate of quinine or cinchonidine may be substituted for the above if a tonic be required, in five grain doses every four to six hours.—*Med. News.*

**Treatment of Sporadic Cholera.**—J. M. French, M. D., in New York Medical Record, says:

My note-book contains the analysis of twenty-five cases of cholera-morbus, occurring during the last three years, in which three different modes of treatment were employed. The cases were of various degrees of severity, some mild and some extremely severe, but all characterized by vomiting and purging, with more or less prostration.

The first plan of treatment consisted in the administration of pepsine, bismuth, alkalies, opiates, and stimulants, by the mouth, with which was frequently combined the application of sinapisms and hot fomentations externally.

The second plan was by means of the rectal injection of starch and laudanum, thirty drops of laudanum being a medium dose; and with this were combined any measures of the first class which might seem desirable.

The third plan consisted in the hypodermic injection of the sulphate of morphia, one-fourth of a grain being the ordinary dose. It was usually unaccompanied by any other treatment.

*Conclusions.*—The first plan of treatment is only adapted to mild and moderate cases, or those which have safely passed the acute stage, as in severe cases no medicine can be retained on the stomach. At best, this plan requires constant watching, and failures are frequent.

The second plan is much more effective, but is liable to need several repetitions on account of inability to retain the injection until absorbed. If not promptly successful patients are apt to lose faith in it, and often object to its repetition. Hence, it is not fully satisfactory.

The third plan is speedy, certain, satisfactory. Except in those cases where all opiates are dangerous, it is also perfectly safe if properly administered. Very seldom does it need repetition. If taken early, it is the only treatment needed. On the whole, I consider the hypodermic injection of morphia as nearly a specific for cholera-morbus as anything in medicine.

**Cellulose as a Dressing.**—Dr. Fischer, of Trieste, has made experiments with cellulose as a dressing to wounds, and has found it, when moistened with warm water or some medicated solution, and afterwards covered with an impervious fabric, to be a most excellent application in all cases where heat and moisture appear to be indicated. Its chief advantages are:

1. It is absolutely free from substances capable of exciting putrefaction.
2. It has a very low specific gravity.
3. It produces neither eczema nor erythema upon the epidermis.
4. It retains moisture and heat perfectly for more than twenty-four hours.
5. It never adheres to granulating wounds on the surface of the skin.
6. It adapts itself perfectly to the outline of the place of application.
7. It is much cheaper than other materials heretofore used for similar purposes.

Dr. Fischer has used, so far, only plain water or weak solution of carbolic acid or iodoform in the case of suppurating buboes, and has obtained uniformly satisfactory results.—*Zeitsch. f. Therap.*

**Commotio Retinæ; or Some of the Effects of Direct and Indirect Blows to the Eye.**—Dr. E. E. Holt, of Portland, Me., (Ophthalmological Society) read a paper in which he reported four cases. In two of these the patient was struck more or less directly by a stick of wood, in one by a round rod or cane, and in the other by a flat piece of coal, striking the forehead, nose and cheek, not hitting the eye itself. In three cases recovery was uninterrupted. In one there was a relapse. Vision was reduced to a perception of light for four days, after which it began to return, and in the course of two weeks became nearly normal. Relapse then occurred and vision sank, but not so low as it had been after the receipt of the injury. Recovery with a perfect eye took place much more slowly than at first. Dr. Holt gave a detailed history of this case, and also of one of the others.

The President remarked that Dr. Holt had reported four cases manifestly dissimilar in character. From a thorough study of the affection referred to, he had become convinced that commotio retinæ was a phenomenon which had been entirely explained upon the supposition of a fissure running through the optic foramen of the orbit, and was almost entirely a mythical supposition by itself.

Dr. Seely thought the subject could not be dismissed so summarily as Dr. Noyes had supposed. He had reported one case which could not be explained away so readily, because a single application of electricity restored a vast amount of vision, and there was no explanation of the extreme lowering of the vision from which the patient suffered.—*N. Y. Med. Record.*

**New Reasons for Woman's Nursing.**—A new element has been discovered by M. Bechamp in woman's milk, hitherto unknown, which, in a striking manner, dissipates the obscurities of the empiricism on certain points of maternal nursing.

The following are the conclusions reached :

1. Woman's milk contains a ferment of a nature to saccharify raw or boiled starch.
2. The specificity of woman's milk is due to the presence of zymase, which makes it preferable to any other.
3. No equivalent can be found in the milk of cows, asses or dogs.
4. The milk of domestic animals may be taken, pure or mixed, in want of woman's milk, but it is never worth as much.
5. Infants should be breast-nursed in preference to all other kinds of feeding.
6. When infants have arrived at the age of taking feculent food, woman's milk may still be useful in aiding in transformation of starch into sugar.—*Paris Medicale.*

**Action of a Mixture of Vapor of Chloroform and Air.**—By PAUL BURT.—Experiments made by him on dogs : 10 gm. of chloroform diluted in 100 lit. of air caused anæsthesia, but 20 gm. of chloroform diluted in 100 lit. of air caused death. With an es-special apparatus he succeeded in proving the effect of a continuous inhalation for 16 hours, of such a mixture.

2 gm. chloroform in 100 lit. of air produced no noteworthy symptom.

4 gm. chloroform in 100 lit. could be inhaled for 9½ hours without any disturbance or lowering of sensibility, except the temperature, which diminished 4–50.

6–7–8 gm. chloroform in 100 lit. of air, effected an appreciable though very slow diminution of sensibility, temp. decreases, and with 30° the animal died.

10 gm. chloroform in 100 lit. of air, rapid anæsthesia, death 2 to 2½ hours. Temp. 33°.

14 gm. in 100 lit. of air, death in 1½ hour.

18 gm., death in 25 minutes.

20 gm. in 100 lit. of air, sudden death.

Temperature falls always in proportion to the duration of the experiment. The action continues to the last moment.

Not all animals are alike sensitive to the effect of chloroform. The following are his conclusions:

Continuous chloroform will always cause death. Animals weakened from a loss of blood succumb sooner.

In order to chloroform helplessly and harmlessly a dog, give at once a toxic dose (12–14 gm. chloroform in 100 lit. of air), but as soon as sleep ensues continue in much smaller doses. (3–6 in 100). —*Gaz. des Hop.—St Louis Med. Jour.*

**Iodine Painting in Small-Pox.**—In 1881 there was admitted to the Konotop hospital a woman suffering from lumbar pain and other prodromal symptoms of small-pox. To satisfy the wish of the patient, Dr. Vetroff painted the whole lumbar region with the tincture of iodine. On the next day the painted region was found covered all over with a variolous rash, while the remaining surface of the body presented only two vesicles. The course of the disease was remarkably mild. Having learned this curious fact, Dr. Bojinski-Bojko (Vratch, No. 1, 1883), when the epidemic of small-pox broke out in his district, began to paint with iodine the anterior surfaces of the thighs in every patient who came under his notice in the prodromal stage of the disease. In all four cases treated in this way the rash was strictly limited to the region painted, and the course of the affection was extremely favorable. An attempt to substitute a sinapism for the iodine gave negative result.—*Med. Times.*

**Arbor-Vitæ.**—Spermatorrhœa is claimed to be finely under the control of this remedy (thuya occiden.) by Dr. Noble, in Therapeutic Gazette, who says he has used it in thirty cases, with but one failure. He uses the homeopathic mother tinct. in doses of 2 to 5 drops three times a day, in conjunction with proper diet, moral control of patient, and other needful accessories. He claims very flattering results to have been derived from this remedy, more than from any other used in the treatment of this affection. —*Ga. Ec. Med. Journal.*

**In Urethral Stricture** "I have," says M. Diday, "in order to avoid confounding it with a spasm and to overcome this if it exists, an infallible method. When the end of the sound is in contact with the coarctated portion of the canal, I suddenly put the following question to the patient: 'How long is it since you have been with a woman?' If it is a simple spasm the sound immediately enters."—*Lyon Medical.*

**Pulse and Temperature in Typhoid Fever.**—M. Malherbe, in a recent These de Paris, remarks that the frequency of the pulse in this disease is not always in proportion with the elevation of temperature. The temperature often becomes very high without a corresponding change in the pulse, and inversely, the pulse may become very much accelerated without any extra elevation of temperature. In any febrile affection where, with a high temperature, the pulse remains almost normal in frequency, typhoid fever should be thought of. The prognosis is not generally bad when the pulse remains at 80 or 90 beats per minute, even when the temperature amounts to 104° or 105°. But when the pulse is very frequent in conjunction with this high temperature, then the prognosis is grave. When, on the other hand, the temperature suddenly falls, while the pulse remains very frequent, the prognosis is equally grave.—*Med and Surg. Rep.*

**Calomel in Diphtheria.**—Dr. Charles S. Miller reports a case of diphtheria in which the breathing was very much embarrassed by the membrane. Calomel in ten-grain doses every hour, until twelve doses were given, was followed by prompt recovery, the membrane being thrown off and showing no tendency to re-form. Neither catharsis nor emesis followed these apparently heroic doses. The case seems strongly corroborative of the claims made by Dr. Reiter in a recent number of Squibb's ephemeris. Dr. Reiter, however, recommended the calomel in the same size doses before the membrane appeared, and to prevent its formation, having little or no faith in this treatment after the patch had formed. We should be very much pleased to receive any report on the use of calomel as above. Dr. Reiter's claims for the drug employed in this manner are too positive to be allowed to pass without subjecting it to a trial.—*Southern Clinic.*

THE discovery of the plax-scindens in oysters, and other shell fish, now that this microbe is known to cause scarlatina, awakens fresh interest in the professional mind. The learned and able Dr. Eklund, of Sweden, hints at the possible vaccinal protection against scarlet fever from the introduction of the schizomycetes which he has found in oysters. It would appear strange if the oyster should supply us with a germ which shall as effectually protect us from scarlatina as that supplied by the cow protects us from the small-pox.—*Med. Times.*

**Good Sight.**—A noted Philadelphia laryngologist, who, on examining a girl with a relaxed uvula and mucous membrane of the throat, concluded that the cause of the difficulty was some uterine trouble, and for which he advised her to place herself under the care of her family physician. Her reply was: "Doctor, if I had known that you could see all the way down, I should not have come to you."—*Exchange.*

**Acute Tonsillitis.**—A German Physician cures acute tonsillitis by the administration of twenty-grain doses of salicylate of soda every four hours. In no case did suppuration ensue.

**Enlarged Tonsils.**—Dr. Sabin, in the Journal of the American Medical Association, says: I notice in the Journal an item in regard to the treatment of enlarged tonsils which put me in mind of a case which I had treated some time ago. I was called to see a little girl, about six years old, who had both tonsils enormously enlarged, so much so that she could hardly breathe, and was becoming pigeon-breasted.

My *modus operandi* was as follows: I took a stick of caustic potash, rolled it in paper, leaving one end bare, placed the bare end on about the middle of the tonsil, held it there about two seconds, withdrew it, waited about a minute, then had her rinse her mouth with vinegar. Repeated treatment twice per week until they reduced to normal size, being careful to apply it in the same place each time. I made, in all, twenty-three applications, treating but one tonsil at a sitting.

I treated her three years ago, and her tonsils have been all right since.

**Cholera Infantum.**—Dr. Walker thinks that Henoch's treatment would be valueless in a vast majority of cases. He recommends in the place of it, the plan adopted by Profs. D. N. Kinsman and Pooley, of Columbus, Ohio. These gentlemen commend injections of ice-water, cold compresses to abdomen, and in addition to this Dr. Pooley advises the use of sub. nit. bismuth in the following doses: He takes enough bismuth to make a tolerable thick cream, using aqua dist. as the vehicle; of this he gives from one-half to one teaspoonful about every four hours, until choleriform discharges cease.

Dr. Walker writes: "This plan of treatment, I believe, is destined to become the treatment par excellence in cases of cholera infantum."—*Ex.*

**The Permanency of Eserine Solutions.**—Dr. Wadsworth spoke of the loss of power in solutions of eserine. Recently he had found a solution which was three years old, and it still worked perfectly well.

Dr. Seely, of Cincinnati, said that for ordinary purposes he preferred an old solution of eserine to a fresh one.—*N. Y. Medical Record.*

**Menorrhagia---Cannabis Indica.**—Four drops of Squibb's fl. extract of cannabis indica, three or four times a day, is an almost certain cure for simple menorrhagia. Generally the preparation as found in the drug stores is too poor in quality to be relied on.—*Ga. Ec. Med. Journal.*

**Effect of Pilocarpin upon Color of the Hair.**—Dr. Pohlman (Buffalo Medical Journal) says: "I have succeeded in demonstrating the capability of pilocarpin to darken the color of the hair in the presence of pigmentary matter, perhaps by a stimulating action on its formation; but that the drug is unable to produce color where pigmentary matter is absent, as shown in the case of experiments upon Albino rabbits.

